# Person-Centered Trauma-Informed Care (PCTIC): A Training Needs Assessment for Home and Community-Based Services in West Virginia

**DOI:** 10.13023/jah.0703.06

**Published:** 2025-09-01

**Authors:** Ranjita Misra, A. Brianna Sheppard, Natalie Wilson, Megan C. Govindan, Matthew Myers, Amanda Acord-Vira, Steven Wheeler, Heather Henderson, Christena Ross, Laura Cooper, Rachel A. Goff

**Affiliations:** West Virginia University; West Virginia University; West Virginia University; West Virginia University

**Keywords:** adverse childhood experiences, Appalachia, HCBS, Home and Community-Based Services, Medicaid, PCTIC, Person-centered trauma-informed care, rural, trauma, workforce training

## Abstract

**Introduction:**

Person-centered, trauma-informed care (PCTIC) is a combined, holistic service model promoting the well-being and empowerment of trauma survivors.

**Purpose:**

This study examined the PCTIC knowledge, skills, and abilities needed by West Virginia (WV) Medicaid Home and Community-Based Services (HCBS) direct service professionals (DSPs) to serve clients that may have experienced trauma.

**Methods:**

Semi-structured interviews were conducted with diverse stakeholders (n = 32) from 19 organizations that provide HCBS services in all 55 counties. Interviews focused on identifying current training, needs and knowledge gaps, and priority areas for additional PCTIC training. ATLAS.ti software was used to manage and code. Validity was established by using multiple data coders, gathering insights from multiple roles, and theory triangulation. Thematic analysis identified training needs and knowledge gaps for PCTIC approaches.

**Results:**

Stakeholders identified knowledge gaps and training needs to address knowledge and empowerment of trauma, positive behavior reinforcement, and access to mental health services. Knowledge gaps included standardized operational definitions related to trauma and PCTIC and the importance of assuring accessibility for evidence-based training for all staff levels. In addition, tailoring for various educational backgrounds and skills was recommended to achieve new performance levels among DSPs. Areas to strengthen current trainings included facilitation of culture change, self-care, listening skills, setting boundaries, and problem-solving/critical thinking.

**Implications:**

Results confirm PCTIC training needs for DSPs to strengthen the WV Medicaid HCBS workforce. However, HCBS training implementation and competency areas should consider factors such as understanding/balancing the training needs and abilities of clinically and non-clinically trained workforce and organizational responsiveness.

## INTRODUCTION

Person-centered trauma-informed care (PCTIC) is a combined, holistic intervention model promoting the well-being and empowerment of trauma survivors.^1^,^2^ Unresolved childhood trauma affects social, academic, and neurobiological development^1^ and may have future negative impacts on adult roles such as parenting and employment, health status, and risk-taking behavior.^3^ The Adverse Childhood Experiences Study identified early initiated drinking and smoking, depression, obesity, suicide attempts, sexually transmitted diseases, illicit drug use, stroke, chronic obstructive pulmonary disease, and cancer as specific areas where trauma experienced in childhood was negatively associated with later life health and well-being.

PCTIC integrates the concepts and principles of trauma-informed care (TIC) and person-centered care (PCC). TIC focuses on trauma awareness and how trauma affects individual behaviors and cognitive development.^1^ TIC is a treatment philosophy based on the effects of traumatic stress and post-traumatic stress disorder (PTSD),^4^ as well as exposure to multiple kinds of traumatic events, especially adverse childhood experiences.^5^,^6^ PCC puts individual needs and goals at the forefront of the healthcare intervention process. TIC has been associated with positive behavioral outcomes and reductions of negative symptoms in psychological disorders like PTSD, depression, anxiety, and attachment anxiety or avoidance.^8^ The adoption of TIC principles has been associated with reducing compassion fatigue (i.e., the physical, emotional and psychological exhaustion that occurs from prolonged exposure to trauma and suffering).^9^ PCC frameworks have successfully reduced agitation and promoted positive behavioral health in patients with dementia,^10^ depression, and anxiety disorder,^11^ and promoted communication and positive outcomes in family planning.^12^ The PCTIC holistic model integrates the psychological and social dimensions of pathology with the biological aspects of disease and lays the foundation for delivery of quality healthcare services.^7^

The PCTIC approach requires service systems to increase professionals’ knowledge of how trauma may impact children, families, and staff in the system and to avoid re-traumatization.^13^ There is a critical need for PCTIC training in West Virginia (WV) since direct serve providers (DSPs) serve Medicaid waiver enrollees who have experienced traumatic events, such as the substance and opioid use epidemic compounded by the COVID-19 pandemic. Home and Community-Based Services (HCBS) DSPs provide PCC to individuals in home and community settings that emphasize individuals’ activities of daily living, enabling members to remain living safely in their communities. While entry-level orientation training is required to prepare, assist, and retain DSPs, current expectations far exceed the training and support provided to properly care for clients experiencing trauma. Providers and staff must be aware of trauma-related symptoms and disorders and how they affect clients.^1^ Identifying signs of secondary traumatization and preventing/ameliorating its effects is necessary regardless of experience level. Providers helping clients must avoid re-traumatization^1^ by not moving too deeply or quickly into trauma material or discounting or disregarding a client’s report of trauma as well as vicarious trauma. An understanding of DSP knowledge and skills is critical for developing competency-based training.

In WV, 54 of 55 counties are mental health professional shortage areas.^14^ There is a paucity of information on necessary PCTIC skills for DSPs providing services to Medicaid waiver enrollees that may have experienced trauma. Such information will offer the opportunity to develop specific skills and advanced-level training to help mitigate staff burnout and improve retention. A statewide needs assessment, described here, was conducted to determine the current status of PCTIC training and identify knowledge gaps and training needs for Medicaid HCBS DSPs.

## METHODS

### Participants

The initial list included 257 agencies and organizations that served waivers and employers of key stakeholders in the state. To ensure adequate representation of the four regions in the West Virginia Department of Human Services (WVDHS) Bureau for Medical Services, all 55 counties, each of the waiver programs, and the agencies/organizations were subdivided into stratum. First, all agencies, their waiver type, and counties served by the agency were compiled into a database. Second, agencies with broader reach (i.e., number of waivers and counties served) were prioritized over those with limited reach. For example, an agency that served four waivers and 55 counties was prioritized over another agency that served only one waiver and four counties. The final subsample of agencies (n=40) selected for stakeholder interviews included all agencies that served three to four waiver programs in at least seven to eight counties (n=7), agencies that served 2 waiver programs in at least 5 counties (n=21), and agencies serving one waiver in at least 15 counties (n=12) in the state of WV. Hence, the organizations selected for stakeholder interviews were representative of the services offered to members/clients in the state. Provider agency leaders, program managers and clinicians, subject matter experts, and DSPs serving HCBS programs (Personal Care, Substance Use Disorder, Children with Serious Emotional Disorders, Aged and Disabled, Traumatic Brain Injury, and Intellectual and Developmental Disorders) were requested to voluntarily participate in a semi-structured needs assessment interview (~45 minutes). The goal was to understand current work and training experiences with PCTIC, the types of clients served, and the identification of needs and preferences regarding training on this specific topic. Thirty-one key stakeholders representing 19 agencies serving all WV counties, regions, and Medicaid HCBS waivers and programs participated in the semi-structured interview. The final composition of the respondents included Subject Matter Experts (n=3), Leadership (n=6), Program Managers and Clinicians (n=13), and Direct Service Professionals (n=10).

### Interview Guide

A semi-structured interview guide was developed by a multidisciplinary team to understand current PCTIC trainings and policies of HBCS waiver programs and services, as well as identify DSPs training needs and preferences, the types of clients served, and demographics. The needs assessment interview guide was based on prior trauma-informed organizational self-assessments.^15^ The guide included 18 questions designed to collect explorative and in-depth information from key stakeholders who have organizational experiences and perceptions of current workforce PCTIC (initial/annual) training, practices, policies, challenges, and organizational cultures that influence behavioral health service providers’ delivery of evidence-based practices to work effectively with trauma survivors. The training needs section used open-ended questions and probes. The content was reviewed by experts, and the interview was pilot tested on two administrators and DSPs. Their feedback was used for minor modifications and questions were tweaked for conversational interviewing prior to conducting stakeholder interviews among a diverse and purposive sample of key stakeholders. This study was reviewed and acknowledged as non-human research subjects research/public health surveillance by the West Virginia University Institutional Review Board (Protocol #2112482700).

### Data Collection

Two trained investigators conducted all Zoom interviews. Participation was voluntary, and stakeholders could skip questions they felt uncomfortable with or were unsure of how to respond. All participants were read a consent statement by one of the investigators at the beginning of the session. Once participants had a chance to ask any questions and agreed to participate, audio recording was enabled for the session, and participants were asked to repeat that they had consented to participate in the study as part of the recorded Zoom interview. Audio from interviews was digitally recorded for transcription in January – March 2022. Prior to each interview, the participants were provided with interview questions, project overview, and assurance their identities would be kept confidential. Interviews were 40–50 minutes. Nineteen interview transcripts and associated notes were analyzed, representing more than 20 interview hours from 31 key stakeholders. While most interviews were for each stakeholder, a few interviews included small groups of two to three individuals from the same agency/organization.

### Data Analysis

ATLAS.ti software was used to manage and code the data.^16^ Thematic analysis consisted of researchers familiarizing themselves with interview transcripts and reflective notes to identify key constructs and define broader themes or needs for PCTIC approaches for DSPs. The qualitative analysis included the identification of key participant organizational characteristics and training needs and knowledge gaps categorized by role: leadership/administration, program managers, and DSPs. The data’s trustworthiness was addressed by creating an audit trail of the transcripts and using a memo process. The memo process included reflective notes from the researchers’ recordings of concepts and their relationships. Reflective notes were de-identified, and inductive codes informed data interpretation. Template analysis, a form of thematic analysis emphasizing the use of hierarchical coding while balancing a high degree of structure in analyzing textual data with the flexibility to adapt it to a particular study’s needs, was used.

The validity of the study was established by using multiple data coders, gathering insights from multiple roles, and theory triangulation. Triangulation of codes occurred by looking for outcomes agreed upon by the PCTIC team coders. Theory triangulation involved using multiple perspectives to interpret data sets. All interviews were coded by three researchers, and all discrepancies were resolved by consensus. Researchers systematically organized data into categories through iterative rounds of data analysis, yielding themes and subthemes. Three rounds of coding ensured distinct concepts and themes were identified, axial coding was completed, and themes were refined and grouped into overarching categories. Lastly, data was refined into meaningful themes that described the range of responses within each theme/subtheme. Eighteen preliminary codes and 114 additional codes were used to identify 35 unique codes, distributed in three categories: challenges (10 codes), training (14 codes), and waivers (nine codes) (Table 1). Four codes (Subject Matter Experts, Leadership, Program Managers, and Direct Service Professionals) were used to identify and categorize interviewee job roles. Each theme and subtheme represented the participants’ voices that presented across multiple interviews, categories (i.e. Challenges, Training, and Waiver Program Characteristics), stakeholder groups (i.e. SME, Leadership, Program Managers, DSPs), and commonalities in current knowledge gaps and PCTIC training needs. Comparative analysis of the transcripts was undertaken to describe commonalities and differences in responses for the key themes within and across categories and stakeholder groups, in consideration of data saturation.

## RESULTS

The key stakeholders represented 19 organizations that provide HCBS services for all six waivers and programs in WV. The agencies represented in the stakeholder interviews reported service areas that collectively encompass all 55 counties in the state, ensuring that perspectives were drawn from HCBS personnel serving both rural and urban regions. This comprehensive representation enhanced the relevance and applicability of our findings to HCBS delivery of PCTIC training needs for individuals with diverse needs in the state. However, only eight interviewees were able to provide a client count.

Themes fit within three categories, including Challenges, Training, and Waiver Program Characteristics (Table 1). Challenges focused on high levels of variation among training, staff attrition, and burnout, as well as access to providers and unintended negative impacts for client participants in the current system. These characteristics were re-emphasized within the categories of existing training and varied experiences among the respondents from WV’s Medicaid *waiver* programs.

Challenges are detailed by stakeholder group (Subject Matter Experts, Leadership, Program Managers and Direct Service Professionals) in Table 2. Challenges converged into 21 primary themes proposed for consideration in training program development and implementation. The multiple stakeholders had differing backgrounds and roles in their organizations. Participants highlighted ensuring that the training is accessible. Stakeholders expressed concerns about staff burnout and the need for support, client access to care, variation in operational definitions of trauma, and training requirements.

Knowledge gaps and training inconsistencies were identified by interviewees (Table 3) and included three themes with 12 subcategories. Knowledge gaps included the need to establish operational definitions related to trauma and PCTIC as it varied between organizations, agencies, and waiver programs, the importance of standardized, evidence-based training for all staff levels, and assuring accessibility for training and service delivery.

Learning outcomes were derived from the qualitative themes for consideration of future PCTIC training (Table 4). These learning outcomes can serve as the foundation of the PCTIC course objectives to address knowledge gaps identified by stakeholders.

Some educational planning considerations included using standardized definitions of trauma (e.g., PCTIC, TIC), employing a person-centered, trauma-informed care strategy, utilizing interactive learning activities, using leadership perspectives to help identify and address challenges for implementing PCTIC training at operational and organizational levels, and collaboration with local resources and agents of change to support PCTIC informed service delivery. Recommendations included balancing emphasized balancing the needs and capabilities of clinical professionals,^17^ other staff members, and family/friend caregivers recognizing their varying abilities to address trauma-related issues. Additional suggestions included using multiple trainers to facilitate training sessions, prioritize in-person training and experiential learning, and promoting organization-wide integration to sustain changes.

### Strengths and Limitations

This study has several strengths, such as the representative diverse stakeholder feedback and comprehensive interview information on training needs and gaps. However, there are several study challenges that were related to conducting interviewers and obtaining responses, and the authors made a solid effort to obtain participation. In addition, while the participants represented 19 organizations that provide HCBS services for all six waivers and programs in all 55 WV counties, smaller organizations with limited reach and services may have different training requirements due to limited staff and resources that may not have been captured in the stratified sampling methodology. Perhaps in a future study, a more structured survey could be conducted to assess training priorities related to topics identified in this current study.

## IMPLICATIONS

This is the first study to systematically examine PCTIC needs and gaps across a diverse sample of provider agency personnel and experts serving multiple HCBS programs in WV. Our findings demonstrate that both within and across HCBS programs and agencies, PCTIC knowledge varied by educational backgrounds among HCBS professionals. The diversity of knowledge concurs with providers’ awareness of the impact of trauma and conceptualization of trauma-informed practice across and within the systems.^18^ Hence, there is an urgent and critical need for effective and sustainable PCTIC training of these professionals to be able to recognize and respond to the needs of trauma survivors. Study findings also highlight the awareness among key stakeholders of the importance of PCTIC training to develop the skills and knowledge they need to provide trauma-informed care and mitigate the negative effects of trauma on the health and well-being of DSPs and the clients they serve. Additionally, findings shed light on training challenges faced by agency personnel to provide a foundation for addressing training needs in the state. However, findings may not be generalizable to organizations outside of WV. Additionally, organizations in the state with limited number of waivers and counties served were excluded; that limits generalizability of the findings to all agencies in the four regions in Bureau for Medical Services. Furthermore, organizational characteristics (e.g., budget size, number of employees, etc.) may impact training needs and gaps and should be explored; this, too, limits the generalizability of the findings. Conducting interviews and obtaining survey responses often present common challenges, and future studies may use a more structured survey to assess training priorities identified in this study. Nonetheless, balancing the training needs and capabilities to address varying knowledge and abilities of the DSPs to address trauma-related issues concurs with prior research.^17^

Barriers and challenges to participation in PCTIC training, or additional training, included consideration of high caseloads for DSPs resulting in a lack of available time, lack of funds to support training attendance, and lack of reimbursement for completing trainings. This is consistent with a previous study that found a lack of conceptual clarity and insufficient funds impede adoption of TIC.^19^ Despite the associated costs of system-wide training implementation, key stakeholders preferred experiential, in-person training over remote or internet-based delivery. Future training should consider what components of online versus in-person training are most beneficial for online learners with varying digital literacy experiences.^20^

The need for operational definitions and standardized training across healthcare service delivery programs transcended across the diverse HCBS programs and stakeholders and concurs with prior literature.^19^ Findings also revealed variability in the definition of trauma and a lack of shared language important to identify trauma in individuals and utilize a trauma-informed approach for service provision. A lack of shared definitions should be considered as a vital component of the training curriculum. Importantly, results showed participants lacked an understanding of the differences between PCTIC and TIC. Hence, inclusion of topics such as a culture of self-care for service providers, listening skills, boundary setting, and problem-solving reflects the workforce needs. This study reinforces previous research for an improved awareness of PCTIC as a holistic approach to providing services.^13^

Some WV Medicaid HCBS programs require training in TIC while other programs do not. A survey by Keesler (2020) explored DSP perspectives of a trauma-informed organizational culture for people with intellectual and developmental disabilities.^9^ The study results showed while organizational practices aligned with TIC, minority DSPs had a more favorable perception, and perceptions did not vary by educational level.^9^ However, providers in this study had diverse educational backgrounds that ranged from doctoral-level trained professionals to family caregivers. Individuals with advanced degrees were more likely to have received training on person-centered and trauma-informed care strategies.

The personalized nature of PCTIC lends itself well to supporting the needs of the HCBS workforce in rural areas. The 2023 Appalachian Regional Commission report highlights the traumatic effects of a decade-long epidemic of substance and opioid use compounded by the COVID-19 pandemic that have led to high rates of DSP turnover and burnout across the states.^21^ An approach that fosters improved provider-client communication, reduced client agitation, improved safety, and increased healthcare professional job satisfaction carries significant benefits to a fragile workforce. The benefits of trauma-informed training programs have been seen in studies among educators who reported positive impacts on attitudes towards trauma-informed care.^22^ Participation in TIC and the implementation of trauma-informed practices has also been associated with improvements in organizational culture and professional satisfaction, including increased awareness and recognition of burnout and secondary traumatic stress among government workers.^23^ However, it is also critical for evaluation of TIC training outcomes as related to stress management, coping and avoiding burnout as DSPs and staff encounter complex and life situations as they encounter and have daily interactions with clients.

A person-centered, trauma-informed approach requires the ability to establish rapport and trust. Including diversity training that involves learning specific terms associated with the region, cultural attitudes, cultural humility, and respect for client autonomy may help providers overcome initial mistrust of professionals and facilitate better therapeutic relationships.^2^

The PCTIC training needs assessment is the first to document WV HCBS workforce training needs for this topic. Feedback from key stakeholders indicated PCTIC training is necessary and will benefit DSPs and the Medicaid enrollees they serve. However, there are numerous considerations to make PCTIC training accessible and appropriate in WV and Central Appalachia. Considerations include balancing needs and abilities of clinical versus other DSP staff, as well as including family/friend caregivers since these diverse professionals have differing abilities to approach trauma-related issues.

Our findings expand the literature by providing additional training factors that facilitate a culture change (e.g., self-care for all professionals, listening skills, boundary setting, and problem-solving/critical thinking). Future studies should examine these needs and gaps in a larger sample size and among DSPs to confirm replicability of findings. Additionally, future studies may include an inquiry about training related to helping staff avoid and manage stress and burnout in their roles given the life situations they encounter with clients of these organizations. While organizational change is a big undertaking for WV, implementing PCTIC training for WV HCBS providers will support successful execution of trauma-informed strategies and initiatives at community, state, and systems levels. Maryland, Tennessee, Michigan, and Wisconsin have successful statewide initiatives for TIC, and Colorado and Oregon have focused on childhood trauma. However, none were driven by system-wide workforce development efforts used in this study.^24^ Our findings provide an important foundation of perceived challenges and knowledge gaps to PCTIC training for the development and implementation of a culturally sensitive curriculum in WV.

**SUMMARY BOX**

**What is already known about this topic?**

The integration of trauma-informed care and person-centered care carries benefits for both clients and providers, including positive behavioral outcomes, reductions of negative symptoms of psychological disorders (like post-traumatic stress disorder, depression, and anxiety) as well as reducing compassion fatigue.

**What is added by this report?**

Our findings provide an important foundation of perceived challenges and knowledge gaps to PCTIC training for the development and implementation of a culturally sensitive curriculum in WV driven by a system-wide workforce development assessment.

**What are the implications for future research?**

Future studies should examine these needs and gaps in a larger sample size of DSPs to confirm replicability of findings and to identify additional targets for organizational change to successfully implement PCTIC training for WV HCBS providers that supports execution of trauma-informed strategies and initiatives at community, state, and systems levels of care. In addition, evaluation of PCTIC training outcomes as related to stress management, coping, and avoiding burnout is critical as these individuals encounter complex and life situations they encounter with clients and daily interactions.

## Figures and Tables

**Table 1:** Theme Identification Derived through Qualitative Analysis

Category	Theme
Challenges	▪ Differences in educational requirements and standards, curriculum design, and delivery vary widely amongst organizations. ▪ Staff shortages and high turnover, exacerbated by population loss, contribute to staff burnout. ▪ Lack of access to DSPs contributes to high caseloads, making person-centered and individualized care challenging due to workforce shortage. ▪ Engagement in the system to access services can be traumatic and cause additional trauma to clients.
Training	▪ Operational definitions are inconsistent amongst all groups, with different definitions used for trauma, person-centered care, and person-centered trauma-informed care. ▪ Access to training and education varies by facility and urban/rural setting. ▪ Desire to engage staff outside of direct-service providers in training to reduce traumatization by staff in the facility. ▪ Lack of staff training related to active listening, problem solving skills, mental health, trauma, traumatic stress, staff self-care, and boundary setting.
Waiver Program Characteristics	▪ Mandatory training is required to facilitate waiver services, and training is incorporated as part of staff orientation. ▪ Education is facilitated by national and local organizations, but there is an overall lack of PCTIC training and understanding of PCTIC vs Trauma-Informed Care. ▪ Compliance to generate revenue is a priority. ▪ Agencies desire more staff, training, and support.

**Table 2:** Challenges Reported by Stakeholders

Group	Theme
Subject Matter Experts (n=3)	▪ Demonstrated need for culturally competent care and staff self-care. ▪ Licensed professionals have additional testing and assessment requirements that vary by field. ▪ In-person delivery of training contributes to networking and support systems of DSPs. ▪ Children need increased support to navigate trauma and prevent further traumatization.
Leadership (n=6)	▪ Population loss, pay compensation, staff burnout and limited access to DSPs with advanced education and training. ▪ Addressing and responding to power struggles between direct service providers and clients dealing with trauma; client autonomy in decision making, positive behavior reinforcement and access to mental health services. ▪ Long wait times to access specific training and access to care. ▪ Pathways for individuals aging out of services. ▪ Lack of evaluation of training that is implemented and inconsistent operational definitions. ▪ Dependence on Medicaid reimbursement.
Program Managers and clinicians (n=13)	▪ Workforce turnover, access to DSPs, and development. ▪ Lack of evidence-based practices related to PCTIC. ▪ Need for experiential learning. ▪ Providers have limited time to complete education due to schedule constraints from providing home services. ▪ Spectrum of educational requirements for staff, clinicians, and DSPs. ▪ Inconsistent operational definitions of trauma. ▪ Need for self-care practices to retain and protect staff.
Direct Service Professionals (n=10)	▪ Need for self-care practices, setting personal and professional boundaries, strategies to preserve mental health. ▪ Lack of feedback loops and assessments on existing training. ▪ Differences in training requirements by agency. ▪ Lack of contact from BMS and ability to share experiences.

**Table 3:** Knowledge Gaps and Training Inconsistencies Identified by Interviewees

Theme	Findings
Operational definitions vary dramatically between organizations, agencies, positions, and waiver programs served	*Inconsistent operational definitions of . . *. ▪ Trauma, socio-economic status, and poverty as a form of trauma, violence and physical abuse, accidents, grief, and conflation amongst terms. ▪ Person-Centered vs Person-Centered Trauma-Informed Care Approaches.
System-wide lack of understanding of operational definitions	*The lack of shared language leads to . . *. ▪ Misunderstanding of PCTIC through all levels of organization and multiple levels of management. ▪ Overtraining by facility and providing training not directly related to waivers. ▪ Unfamiliarity in understanding training needs by DSPs, clinicians, program managers. ▪ Perception of training effectiveness.
Current training is inconsistent by facility, sponsoring organization, and waiver.	*Knowledge gaps are intensified by . . *. ▪ Various requirements for waiver providers. ▪ High caseloads of DSPs that prevent conversations to help address person-centered training and needs. ▪ Lack of formal mechanism for reimbursement/billing for DSPs to attend training opportunities. ▪ Disproportionate training amongst staff (individuals with formal education are already getting the training, while less credentialed practitioners may receive none). ▪ Impacts of COVID-19 and inability to meet face-to-face with clients and for training. ▪ Accessibility of training by DSPs vs clinical care providers.

**Table 4:** Learning Outcomes to be Addressed in PCTIC Training

Outcome Verb	Findings
Create	▪ Safety and crisis plan for clients. ▪ Person-centered trauma-informed care strategies.
Analyze	▪ Psychosocial and mental problems by conducting appropriate assessment(s). ▪ Difficult behaviors through a trauma-informed lens. ▪ Management of triggers (e.g., helplessness, rage, sadness, terror, etc.). ▪ Secondary traumatic stress/trauma to for the development of self-care plans that alleviate its effects.
Apply	▪ Listening skills and self-care practices. ▪ Evidence-based interventions with the target population. ▪ De-escalation strategies to prevent crisis. ▪ Strategies for solving problems on individual, family, and community levels.
Define	▪ Effects of traumatic stress on the brain and body. ▪ Treatment approaches for medically unexplained somatic pain. ▪ Impacts of working with trauma survivors on DSPs and staff. ▪ The relationship between mental health, trauma, and traumatic stress.
Remember	▪ Operational definitions ▪ Trauma (physical, social) ▪ Person-Centered Care ▪ Person–Centered Trauma-Informed Care ▪ Setting and maintaining personal boundaries with clients. ▪ Local service delivery landscape to inform expectations, duration of treatment, and client attitudes towards intervention.
